# The Psychological Well-Being of Southeast Asian Frontline Healthcare Workers during COVID-19: A Multi-Country Study

**DOI:** 10.3390/ijerph19116380

**Published:** 2022-05-24

**Authors:** Irene Teo, Gayathri Devi Nadarajan, Sean Ng, Adithya Bhaskar, Sharon C. Sung, Yin Bun Cheung, Fang Ting Pan, Ali Haedar, Faith Joan Gaerlan, Sheue Fen Ong, Sattha Riyapan, Son Ngoc Do, Chinh Quoc Luong, Vijaya Rao, Lin Min Soh, Hiang Khoon Tan, Marcus Eng Hock Ong

**Affiliations:** 1 Programme in Health Services & Systems Research, Duke-NUS Medical School, Singapore 169857, Singapore; sharon.sung@duke-nus.edu.sg (S.C.S.); yinbun.cheung@duke-nus.edu.sg (Y.B.C.); marcus.ong@duke-nus.edu.sg (M.E.H.O.); 2 Lien Centre for Palliative Care, Duke-NUS Medical School, Singapore 169857, Singapore; sean.ngyw@duke-nus.edu.sg (S.N.); adithya.bhaskar.ab@gmail.com (A.B.); ft_pan@hotmail.com (F.T.P.); 3 Department of Psychosocial Oncology, National Cancer Centre Singapore, Singapore 169610, Singapore; 4 Department of Emergency Medicine, Singapore General Hospital, Singapore 169608, Singapore; gmsgdn@nus.edu.sg; 5 SingHealth Duke-NUS Global Health Institute, Singapore 169857, Singapore; vijaya.rao@singhealth.com.sg (V.R.); tan.hiang.khoon@singhealth.com.sg (H.K.T.); 6 Department of Developmental Psychiatry, Institute of Mental Health, Singapore 539747, Singapore; 7 Centre for Quantitative Medicine, Duke-NUS Medical School, Singapore 169857, Singapore; 8 Centre for Child Health Research, Tampere University, 33100 Tampere, Finland; 9 Saiful Anwar General Hospital, Faculty of Medicine Universitas Brawijaya, Kota Malang 65145, Jawa Timur, Indonesia; haedaryahya@yahoo.com; 10 Philippine College of Emergency Medicine and University of the Philippines—Philippine General Hospital, Metro Manila 1000, Philippines; drfjmgaerlan@gmail.com; 11 Emergency & Trauma Department, Hospital Sultanah Bahiyah, Kedah 05460, Malaysia; sheuefen84@yahoo.com; 12 Department of Emergency Medicine, Faculty of Medicine Siriraj Hospital, Mahidol University, Bangkok 10700, Thailand; sattha.riy@mahidol.ac.th; 13 Center for Critical Care Medicine, Bach Mai Hospital, Hanoi 100000, Vietnam; sonngocdo@gmail.com; 14 Department of Emergency and Critical Care Medicine, Hanoi Medical University, Hanoi 100000, Vietnam; luongquocchinh@gmail.com; 15 Faculty of Medicine, School of Medicine and Pharmacy, Vietnam National University, Hanoi 100000, Vietnam; 16 Center for Emergency Medicine, Bach Mai Hospital, Hanoi 100000, Vietnam; 17 Singhealth International Collaboration Office, Singapore 168753, Singapore; 18 Yale-NUS College, Singapore 138527, Singapore; linmin.soh@u.yale-nus.edu.sg; 19 Division of Surgery and Surgical Oncology, Singapore General Hospital and National Cancer Centre Singapore, Singapore 169610, Singapore

**Keywords:** healthcare worker, psychological wellness, Asia, COVID-19, anxiety, depression, burnout

## Abstract

Objectives: This study examined the prevalence of anxiety, depression, and job burnout among frontline healthcare workers (HCWs) across six Southeast Asian countries (Indonesia, Malaysia, Philippines, Singapore, Thailand, Vietnam) during the COVID-19 pandemic in 2021. We also investigated the associated risk and protective factors. Methods: Frontline HCWs (N = 1381) from the participating countries participated between 4 January and 14 June 2021. The participants completed self-reported surveys on anxiety (GAD-7), depression (PHQ-8), and job burnout (PWLS). Multivariate logistic regressions were performed with anxiety, depression, and job burnout as outcomes and sociodemographic and job characteristics and HCW perceptions as predictors. Results: The average proportion of HCWs reporting moderate anxiety, moderately severe depression, and job burnout across all countries were 10%, 4%, and 20%, respectively. Working longer hours than usual (Odds ratio [OR] = 1.82; 3.51), perceived high job risk (1.98; 2.22), and inadequate personal protective equipment (1.89; 2.11) were associated with increased odds of anxiety and job burnout while working night shifts was associated with increased risk of depression (3.23). Perceived good teamwork was associated with lower odds of anxiety (0.46), depression (0.43), and job burnout (0.39). Conclusion: Job burnout remains a foremost issue among HCWs. Potential opportunities to improve HCW wellness are discussed.

## 1. Introduction

**Figure 1:**
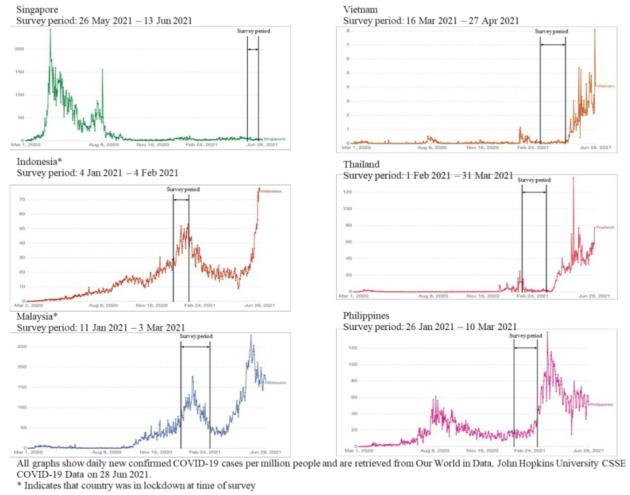
Epidemic curves of COVID-19 in studied country sites.

The ongoing COVID-19 pandemic has placed immense pressure on global healthcare systems. Many Southeast Asian countries adopted strict border controls and stringent public health measures [1], including Indonesia, Malaysia, Philippines, Singapore, Thailand, and Vietnam, which allowed control of the initial waves of infection [2]. However, many struggled to contain the subsequent surges attributed to more virulent and contagious variants (e.g., Delta, which became the dominant strain in 2021). Refer to Figure 1. The situation was exacerbated by sluggish vaccine uptakes [3] despite these countries having fairly robust healthcare systems [4].

The COVID-19 pandemic has also produced many challenges faced by frontline healthcare workers (HCWs), particularly those working in emergency settings, including doctors and nurses in emergency departments and pre-hospital services providers (paramedics, emergency medical technicians [EMTs]), because of their roles in being the first responders to COVID-19 without the benefit of knowing if individuals are infected. Some of these challenges include caring for patients with inadequate resources and personal protective equipment [5,6], taking on increased personal infection risk to provide patients with optimal care [6], and erratic shift work [7]. Some frontline HCWs also report increased stigmatization due to their proximity in working with infected individuals and feelings of social isolation as many choose to live away from their families for fear of transmission to their loved ones [8].

An important focus of the COVID-19 literature has been on the effects of the pandemic on HCW psychological well-being [9,10,11]. Findings from recent systematic reviews indicate that 17% to 45% of frontline HCWs report suffering from anxiety and depression [12,13,14], with worse symptoms experienced by those with more patient contact (e.g., nurses) [12]. Similar patterns were observed for job burnout (5% to 50%) [15,16,17], underlining the psychological sequelae felt by HCWs during this pandemic. There is some evidence, however, that the psychological distress experienced by HCWs in Asia may be lower than that of their Western counterparts [14], suggesting that Asian HCWs may be experiencing the pandemic differently. It may also be under-reported in the Asian context due to the stigma of mental illness prevalent in Asia [18].

To better understand the psychological distress faced by HCWs, risk and protective factors are important to consider. The literature suggests that demographic, occupational, and healthcare worker perceptions are associated with HCW outcomes. A prior study of HCWs in Singapore in 2020 found that working normal hours, perceived good teamwork, and feeling appreciated at work were associated with lower anxiety, stress, and burnout [19]. 

This study had two aims. First, we examined the prevalence of anxiety, depression, and job burnout among healthcare workers in Southeast Asia. We also sought to assess the associated risk and protective factors with these outcomes. We hypothesized that certain job characteristics (occupation, length of career, managerial role, work location, exposure to COVID-19 patients, work night shifts) and HCW perceptions (job risk, working longer hours than usual, adequacy of PPE, clarity of work protocols, and teamwork) would be associated with anxiety, depression, and job burnout controlling for country sites.

This is one of the few studies looking at the wellness of Southeast Asian emergency frontline workers during COVID-19. Findings from this study are important as they will help us understand the protective and risk factors associated with anxiety, depression, and burnout amongst Southeast Asian emergency frontline healthcare workers and eventually help with the development of materials as part of the training framework to prepare for such pandemics. Psychological well-being is an essential element of pandemic preparedness, which has been overlooked historically.

## 2. Materials and Methods

### 2.1. Study Design

This cross-sectional study utilized a convenience sample of HCWs from 6 Southeast Asian countries: Indonesia, Malaysia, Philippines, Singapore, Thailand, and Vietnam, during the COVID-19 pandemic. Self-reported data were collected from 4 January 2021 to 14 June 2021 (refer to Figure 1 for details by country). 

### 2.2. Participants and Data Collection

Country representatives of the Pan Asian Resuscitation Outcomes Study (PAROS) research network [20] and the Asian Association for Emergency Medical Services (AAEMS) were contacted and invited to participate in the study. Most of the Asian countries are active within the AAEMS and the PAROS networks, and the people involved have an EMS or Emergency Care Services background. They work either in the ambulance services or the emergency department or its equivalent within the health system. They may range from Emergency Medical Technicians (EMTs) to Paramedics, to Emergency specialists to doctors trained in Emergencies and even doctors not formally trained in emergencies but who work in the emergency department or the pre-hospital setting. For the survey, the participants included those sent to the emergency department as augmented manpower to help with the COVID situation. Participating country representatives sent out emails to their frontline healthcare workers. Participation was voluntary, and healthcare workers who were interested accessed the study through a web link or QR code. The survey was hosted on Qualtrics and made available in English and the main local languages of the participating countries. 

### 2.3. Measures

The study utilized validated instruments whenever possible and questions developed by the study investigators. Where necessary, the instrument items were professionally translated from English into the primary language of each study site and then back-translated into English. The original and back-translated English versions were compared, and reconciliations were made. Further revisions were made based on feedback from the country representatives. The primary outcomes in this study were anxiety, depression, and job burnout.

*Anxiety* was measured using the Generalized Anxiety Disorder (GAD-7) scale [21], a scale with supported reliability and validity as a measure of anxiety in the general population [22]. The 7 items were summed to obtain a score ranging from 0–21. The recommended threshold score of ≥10, corresponding to moderate anxiety, was used [21]. The cut-off score of ≥10 has been similarly used in peer-reviewed publications from the included countries [23,24,25,26,27,28]. The internal consistency of the measure in this study is high, with Cronbach’s α = 0.91.

*Depression* was measured using the Patient Health Questionnaire (PHQ-8). The 8 items were summed to obtain a score ranging from 0 to 24. The recommended threshold score of ≥15 (i.e., moderately severe depression) was used to indicate the presence of depressive symptoms [29]. The internal consistency of the measure in this study is high, with Cronbach’s α = 0.91. The scale has been used in our countries of interest [30,31,32,33,34,35].

*Job burnout* was measured using a one-item burnout question from the Physician Work Life Scale (PWLS) that instructs respondents to define burnout for themselves: “Overall, based on your definition of burnout, how would you rate your level of burnout?”. The response ranged from “I enjoy my work, I have no symptoms of burnout” (1) to “I feel completely burned out and often wonder if I can go on. I am at a point where I may need some changes or may need to seek some sort of help” (5). A score ≥ 3 indicated the presence of job exhaustion [36]. The non-proprietary measure has been used with a range of HCWs, including doctors, nurses, and allied health professionals. 

*Job characteristics.* Participants reported their occupation, work location, and number of working years in their healthcare career. The responses to the following questions were coded “yes”/“no”: whether they had a managerial role and worked night shifts in the past month. Exposure to COVID-19 was assessed by “How often does your job require you to come in contact with suspected/confirmed COVID-19 patients?” with the response options being “not at all”, “occasionally”, and “daily”. 

*HCW perceptions.* Perceived job risk was assessed using the item “I feel that my job puts me at great risk of exposure to COVID-19” where responses ranged from “strongly agree” to “strongly disagree” on a 6-point scale that was later recoded into a binary variable (high risk vs. low risk) [37]. Working longer than usual hours in the past month, adequacy of PPE at the workplace, clarity of work policies/protocols, and good teamwork at the workplace were assessed as yes/no questions. 

### 2.4. Data Analysis

Demographic descriptive data were summarized using means and standard deviations for continuous variables and percentages for categorical variables. Subsequently, separate multivariate logistic regression models were run with respective burnout, anxiety, and depression as the outcomes of interest and job characteristics (occupation, length of career, managerial role, work location, exposure to COVID-19 patients, working night shifts) and HCW perceptions (working longer hours than usual, high job risk, inadequate PPE, clear work protocols, and good teamwork) as the predictors. All models were controlled for country with Singapore as the reference category. Odds ratios (*OR*) with *p*-values are presented. Analyses were conducted using STATA v16.1.

## 3. Results

A total of 1381 HCWs responded to the survey and were included in the analyses. The sample consisted of doctors (37%), nurses (35%), and EMTs (11%) and others that comprised other healthcare workers, including allied health professionals and hospital administrative staff. The average age of study respondents was 35.61 (*SD* = 9.14). The majority of the sample were female (60%) and married (64%). The plurality of the study respondents worked primarily in the emergency department (39%) and reported occasional contact with COVID-19 patients (47%). The plurality also reported working night shifts (39%) and working longer hours than usual (47%). The majority of respondents perceived their job to be at high risk for contracting COVID-19 (80%) while also reporting adequate PPE measures at work (86%), clear work protocols (61%), and good teamwork (66%). Refer to Table 1 and Table 2 for further details.

### 3.1. Prevalence of Anxiety, Depression, and Job Burnout

Across the countries, the average proportion of healthcare workers reporting moderate anxiety was 10% (GAD-7 *M* = 4.35, *SD* = 4.19), moderately severe depression 4% (PHQ-8 *M* = 4.34, *SD* = 4.54), and job burnout 20% (PWLS *M* = 2.01, *SD* = 0.87). HCWs in Singapore reported the highest (21%, 9%, 39%), whilst HCWs in Vietnam (4%, 2%, and 6%) reported the lowest proportions of anxiety, depression, and job burnout, respectively. Refer to Table 2 for a breakdown by country.

### 3.2. Predictors of Anxiety, Depression, and Job Burnout

Table 3 presents factors associated with anxiety, depression and job burnout. Perceiving high job risk (odds ratio OR] = 1.98, 95% CI [1.06; 3.69], 2.22, [1.34; 3.65]), working longer hours than usual (1.82, [1.06; 3.69], 3.51 [4.26; 5.02]), and perceived inadequacy of PPE (1.89, [1.10; 3.26], 2.11 [1.36; 3.26]) were associated with higher odds of anxiety and job burnout, respectively. Working night shifts was associated with higher odds of depression (3.23, [1.57; 3.20]).

Perceiving good teamwork in the workplace was associated with lower odds of anxiety (0.46, [0.30; 0.70]), depression (0.43, [0.23; 0.79]), and job burnout (0.39, [1.70; 3.67]). Being a nurse (compared to a physician; 0.61, [0.41; 0.92]) and having a longer career in healthcare (0.97, [0.79; 1.66]) were associated with lower job burnout as well.

## 4. Discussion

This study examined the prevalence of important psychological outcomes (anxiety, depression, job burnout) among frontline HCWs in six Southeast Asian countries. Our findings indicated that a small but significant proportion of respondents reported psychological morbidity; job burnout rates were found to be the highest across the countries, followed by anxiety and depression. Our findings suggest that prevalence varied across the countries, where Singaporean HCWs reported the highest rates consistently, while those from Vietnam reported the lowest rates. A potential explanation of this discrepancy, which also generally explains the variation among the countries, is that COVID-19 infection rates in the community (reflecting the stress on the healthcare system) of the given country at the time of the study. Singapore was in a ‘heightened alert’ phase (equivalent to a partial lockdown) [38] in dealing with the Delta surge, while Vietnam was in a relatively stable state with low infection numbers [39]. 

It is also interesting to note that global mental health estimates indicated that, similar to the average of 4% in this study, pre-COVID-19 depressive symptoms in the countries of interest ranged from 3.3% to 4.6% [40]. However, anxiety levels, which averaged 10% in this study, were found to be significantly higher than the pre-COVID-19 estimates of between 2.2% and 4.9% [40]. While noteworthy as they suggest that additional focus should be placed on addressing the anxiety of HCWs, these findings are unsurprising when taken in the context of the pandemic. Given the job-associated risks of being a HCW, including increased risks of infection, concerns regarding the inadequacy of safety equipment (which we discuss as a risk factor later), and fears of COVID-19 transmission to loved ones, it is understandable why a frontliner would face higher levels of anxiety related to the pandemic, but not necessarily depression.

Generally, the proportion of HCWs reporting psychological distress in this study was lower than those found in prior international COVID-19 research [12,13,14,16]. There may be several reasons for these discrepancies. First, it is possible that the relatively low psychological morbidity rates among the HCWs reflect the low infection rates in the countries of interest [41]. It is also possible that the healthcare systems examined were robust enough to mitigate the stressors faced by HCWs during the pandemic. The healthcare workers involved in this study were emergency frontline workers who may be more resilient/psychologically prepared compared to HCWs in other departments. There is also a cultural/social expectation for Asians to be “strong” and to “save face” [42], which may have prevented them from reporting symptoms of distress. 

In examining the risk and protective factors associated with HCW outcomes, we found that longer than usual working hours and perception of high risk from COVID-19 and inadequacy of PPE were associated with higher odds of burnout and anxiety. These results are in line with past research [6,7,19] and underscore the importance of sustaining a healthy work–life balance in the workplace (particularly during stressful periods). Working night shifts was associated with higher odds of depression, which is also supported by the prior literature showing the association between disruptions in circadian rhythms from shift work and mood disorders [43]. 

Some practical implications include respecting work-time boundaries and discouraging a culture of overtime work. However, this may be challenging during a crisis period, such as during the COVID pandemic. Perhaps communicating to prepare the workforce for longer work hours as well as communicating the risks of such a crisis and measures taken to mitigate these risks may help to prepare HCWs. Our findings also highlight the importance of being aware of HCW sentiments and perceptions of COVID-19 risks (including adequacy of PPE) so that HCW misconceptions or worries may be addressed. For instance, the Singapore General Hospital emergency department [44], in the initial months of the pandemic, distributed information pamphlets to staff and their families to provide information on the risks they faced on the frontlines of the pandemic and the steps that have been taken and can be taken to ensure the safety of HCWs and their families.

We also found some protective factors associated with reduced psychological distress. Perceived good teamwork was associated with lower odds of burnout, anxiety, and depression, while having more years of working experience and being a nurse were associated with lower burnout. These findings suggest cultivating a culture of teamwork in the workplace is important in buffering work-related stress. While the majority of participants across all countries in this study perceived that their team worked well together, the inclusion of more teambuilding initiatives can further strengthen the feeling of camaraderie among HCWs. Buddy systems, where junior HCWs are partnered with senior colleagues who have experience can also be potentially useful. 

Our finding that nurses (compared to doctors) had lower odds of burnout contradict the extant literature [45]. While the reason for this discrepancy is unclear, it is possible that the sample in this study, which consists mostly of those who work in emergency settings, is different from prior studies that typically include HCWs from the larger hospital; potentially doctors in emergency settings typically experience more job pressure or nurses in emergency settings tend to be more experienced and capable of handling work pressures. It is also plausible that differences may also be due to the different healthcare systems examined in both studies.

These findings above are important preparations for future crises. Emergency frontline workers have traditionally been trained to manage disasters and medical emergencies. However, little attention has been given to the psychological well-being, psychological first aid, and resilience of frontliners. Findings from this and other studies can help to develop such materials to train the emergency healthcare workforce. For example, the awareness that teamwork and effective communication are protective factors that support a team-based system of working during such a pandemic. There can also be a framework to ensure adequate and effective communication by the emergency system to ease the workers’ fears of inadequate PPE and contracting the illness. This may even include offering to address the workers’ family members when communicating about the risks of a pandemic such as this. Particular emphasis can also be placed on inexperienced frontline workers as they may be most susceptible to pandemic-related workplace stressors. This and other studies reinforce the impact of the pandemic on the psychological state of our frontline workers; hence perhaps, there should be a separate workgroup to support the well-being of the staff as part of pandemic preparedness.

Our results have to be taken in the context of several limitations. First, our findings may not be generalizable to all HCWs in the countries examined as participants were recruited through specific networks and depended on the convenience/snowball sampling method. Participation was voluntary, and there may have been self-selection bias. Our study design, which was cross-sectional, did not allow for the establishment of causation or prediction. It must also be noted that job burnout was measured using an ultra-brief one-item measure. Nonetheless, our findings are an important contribution to the literature and can serve as the foundation for healthcare policies targeted at enhancing the well-being of emergency frontline workers.

## 5. Conclusions

Our study utilized unique data from several countries in the Southeast Asian region to understand the psychological well-being of HCWs, and we found that a small yet significant proportion of participants reported anxiety, depression, and symptoms of job burnout. Job burnout appears to be the foremost issue among healthcare workers, suggesting that this aspect of HCW wellness is important to address among frontline HCWs. Our findings provide a cursory examination across different Southeast Asian countries during a serious pandemic, and future studies are encouraged to build upon our findings to better understand long-term HCW psychological outcomes.

## Figures and Tables

**Table 1:** Sociodemographic and Job Characteristics (N = 1381).

Study Sites Sample Size	Indonesia n = 368	Malaysia n = 131	Philippines n = 54	Singapore n = 276	Thailand n = 128	Vietnam n = 424	Total n = 1381
	M (SD)/N (%)						
***Sociodemographic Characteristics***							
**Age** (mean, SD)	35.50 (7.97)	32.62 (7.71)	39.44 (9.66)	38.05 (11.91)	35.86 (9.59)	34.47 (7.60)	35.61 (9.14)
**Gender** (female)	211 (58%)	46 (36%)	27 (50%)	221 (80%)	95 (74%)	227 (54%)	827 (60%)
**Marital status**							
Single	84 (23%)	50 (38%)	26 (48%)	121 (44%)	88 (69%)	96 (23%)	465 (34%)
Married	274 (74%)	77 (59%)	25 (46%)	150 (54%)	35 (27%)	316 (75%)	877 (64%)
Divorced/widowed	10 (3%)	4 (3%)	3 (6%)	5 (2%)	5 (4%)	12 (3%)	39 (3%)
**Vaccinated at the time of survey**	0 (0%)	0 (0%)	0 (0%)	241 (87%)	1 (1%)	111 (26%)	353 (26%)
							
***Job Characteristics***							
**Occupation**							
Doctor	202 (55%)	44 (34%)	31 (57%)	36 (13%)	34 (27%)	159 (38%)	506 (37%)
Nurse	129 (35%)	9 (7%)	11 (20%)	130 (47%)	67 (32%)	131 (31%)	477 (35%)
EMTs	0 (0%)	29 (22%)	9 (17%)	0 (0%)	13 (10%)	105 (25%)	156 (11%)
Others	37 (10%)	49 (37%)	3 (6%)	110 (40%)	14 (11%)	29 (7%)	242 (18%)
**Length of career** (years)	10.74 (7.74)	8.88 (6.78)	10.64 (7.90)	14.08 (11.23)	12.15 (9.77)	10.05 (6.98)	11.16 (8.63)
**Managerial role**	166 (45%)	61 (47%)	33 (61%)	80 (29%)	64 (50%)	112 (26%)	516 (37%)
**Work location**							
Emergency Department	142 (39%)	79 (60%)	35 (65%)	29 (11%)	106 (83%)	147 (35%)	538 (39%)
Pre-hospital/Ambulance service	4 (1%)	22 (17%)	12 (22%)	0 (0%)	13 (10%)	104 (25%)	155 (11%)
COVID Ward/ICU	43 (15%)	8 (6%)	1 (2%)	21 (8%)	2 (2%)	145 (34%)	231 (17%)
Non-infectious/Clean wards	129 (35%)	14 (11%)	4 (7%)	226 (82%)	6 (5%)	23 (5%)	402 (29%)
Community care/recovery	3 (10%)	8 (6%)	2 (4%)	0 (0%)	1 (1%)	4 (1%)	52 (4%)
**Exposure to COVID-19 patients**							
Not at all	33 (9%)	11 (8%)	3 (6%)	107 (39%)	8 (6%)	154 (36%)	316 (23%)
Occasional	159 (43%)	43(33%)	27 (50%)	119 (43%)	88 (69%)	218 (51%)	654 (47%)
Daily	158 (43%)	74 (56%)	19 (35%)	50 (18%)	28 (22%)	43 (10%)	372 (27%)
Others	17 (5%)	3 (2%)	5 (9%)	0 (0%)	4 (3%)	(2%)	38 (3%)
**Working night shifts**	73 (20%)	52 (40%)	15 (28%)	104 (38%)	63 (49%)	209 (49%)	516 (39%)

*Frequencies may not add up to total sample due to missing data.*

**Table 2:** Healthcare Worker Psychological Outcomes and Perceptions (N = 1381).

Study Sites Sample Size	Indonesia n = 368	Malaysia n = 131	Philippines n = 54	Singapore n = 276	Thailand n = 128	Vietnam n = 424	Total n = 1381
	M (SD)/N (%)						
**Anxiety (GAD-7)** Mean (SD)	4.49 (3.97)	4.74 (4.25)	5.85 (5.08)	5.98 (5.13)	4.45 (3.44)	2.84 (3.15)	4.35 (4.19)
Score ≥ 10	36 (10%)	13 (10%)	9 (17%)	57 (21%)	10 (8%)	15 (4%)	140 (10%)
**Depression (PHQ-8)** Mean (SD)	4.34 (4.19)	5.39 (4.88)	5.30 (5.46)	5.86 (5.43)	4.38 (3.55)	2.88 (3.71)	4.34 (4.54)
Score ≥15	11 (3%)	10 (8%)	5 (9%)	24 (9%)	2 (2%)	7 (2%)	59 (4%)
**Job burnout (PWLS)** Mean	1.87 (0.85)	2.08 (0.89)	2.15 (0.76)	2.49 (1.00)	2.13 (0.73)	1.74 (0.66)	2.01 (0.87)
Score ≥ 3	66 (18%)	32 (24%)	13 (24%)	108 (39%)	31 (24%)	27 (6%)	277 (20%)
High job risk	336 (91%)	118 (90%)	44 (81%)	173 (63%)	110 (86%)	319 (75%)	1100 (80%)
Worked longer hours than usual	155 (42%)	76 (58%)	23 (43%)	131 (47%)	53 (41%)	211 (50%)	649 (47%)
Inadequate PPE	52 (14%)	13 (10%)	6 (11%)	16 (6%)	39 (30%)	67 (16%)	193 (14%)
Clear work protocols	182 (49%)	67 (51%)	32 (59%)	177 (64%)	61 (48%)	321 (76%)	840 (61%)
Good teamwork	241 (65%)	76 (58%)	38 (70%)	181 (66%)	99 (77%)	272 (64%)	907 (66%)

*Frequencies may not add up to total sample due to missing data.*

**Table 3:** Factors Associated with Anxiety, Depression, and Job burnout.

	Anxiety		Depression		Job Burnout	
	*OR*	*CI*	*OR*	*CI*	*OR*	*CI*
**Occupation**						
Doctor *(ref)*						
Nurse	0.80	0.48; 1.32	0.95	0.44; 2.07	0.61 *	**0.41; 0.92**
EMTs	1.10	0.44; 2.78	2.62	0.72; 9.60	0.85	0.39; 1.87
Others	0.63	0.32; 1.24	1.44	0.58; 3.57	0.83	0.50; 1.40
**Length of career**	0.98	0.96; 1.01	0.97	0.92; 1.02	0.97 *	**0.79; 1.66**
**Managerial role**	0.73	0.44; 1.19	1.15	0.56; 2.34	1.15	0.79; 1.66
**Work location**						
Emergency department	1.29	0.69; 3.40	1.36	0.54; 3.44	1.23	0.74; 2.07
Pre-hospital/Ambulance service	0.65	0.19; 2.21	0.78	0.15; 3.96	0.89	0.36; 2.22
COVID ward/ICU	0.67	0.30; 1.49	0.62	0.19; 2.07	1.09	0.58; 2.05
Community care/Recovery	1.14	0.35; 3.76	3.69	0.79; 17.19	1.44	0.55; 2.75
						
*Healthcare worker perceptions*						
**Exposure to COVID-19 patients**						
Occasionally	0.82	0.47; 1.44	0.90	0.41; 1.95	0.85	0.52; 1.38
Daily	1.22	0.64; 2.30	1.10	0.46; 2.59	1.38	0.80; 2.37
Others	0.62	0.14; 2.74	1		0.87	0.24; 3.16
**Work night shifts**	1.44	0.89; 2.32	3.23 **	**1.57; 3.20**	1.21	0.83; 1.76
**High job risk**	1.98 *	**1.06; 3.69**	1.79	0.79; 4.08	2.22 **	**1.34; 3.65**
**Working longer hours than usual**	1.82 *	**1.20; 2.75**	1.63	0.83; 3.20	3.51 **	**2.46; 5.02**
**Inadequate PPE**	1.89 *	**1.10; 3.26**	2.05	0.92; 4.55	2.11 **	**1.36; 3.26**
**Clear work protocols**	1.16	0.75; 1.80	1.65	0.85; 3.22	1.31	0.92; 1.85
**Good teamwork**	0.46 **	0.30; 0.70	0.43 *	**0.23; 0.79**	0.39 **	**1.70; 3.67**
						
**Control (countries)**						
Indonesia	0.28 **	**0.14; 0.55**	0.26 *	**0.09; 0.73**	0.14 **	**0.08; 0.25**
Malaysia	0.20 **	**0.06; 0.45**	0.31 *	**0.10; 0.93**	0.12 **	**0.06; 0.26**
Philippines	0.49	0.16; 1.49	0.75	**0.16; 3.54**	0.28 **	**0.11; 0.70**
Thailand	0.18 **	**0.06; 0.51**	0.07 *	**0.01; 0.45**	0.27 **	**0.13; 0.56**
Vietnam	0.10 **	**0.04; 0.24**	0.13 **	**0.36; 0.45**	0.04 **	**0.02; 0.08**

OR = Odds ratio. ** *p* < 0.01. * *p* < 0.5.

## Data Availability

Individual participant data are not publicly available because of the sensitive nature of mental health information of healthcare work. The study metadata, study protocol and analyses code will be made available upon reasonable request. All requests should be directed to irene.teo@duke-nus.edu.sg. Every request will be reviewed by our IRB and the requestor will need to sign a data access agreement once approval has been given.
